# A Patient-Centered Perspectives and Future Directions in AI-powered Teledentistry

**DOI:** 10.15190/d.2024.18

**Published:** 2024-12-31

**Authors:** Richa Kaushik, Ravindra Rapaka

**Affiliations:** ^1^Wayne State University, Detroit, Michigan; ^2^University of Connecticut, Storrs, Connecticut

**Keywords:** Teledentistry, artificial intelligence (AI), diagnostic accuracy, personalized treatment planning, patient-centered care, data privacy.

## Abstract

This scoping review investigates the integration of AI into teledentistry with a focus on patient-centered perspectives and future directions. Teledentistry has progressed rapidly in the COVID-19 pandemic period, providing remote dental care by means of digital communication technologies.The introduction of AI has made diagnosis more precise, treatment planning more personalized, and processes more efficient and have also made dental services better accessible to the underserved. AI algorithms help in early diagnosis of dental issues, provides customized treatment plans, and improve patient outcomes. Despite the advantages, still many challenges exist. These are ethical concerns, data privacy issues, and regulatory hurdles that prevent widespread adoption. Use of AI in dental settings results in patients having mixed sentiments surrounding trust and data security arising out of fear of having   reduced   personal interactions with providers. Additionally, AI driven teledentistry is not validated in large scale clinical setting and cost effectiveness assessment which undermines scalability. This review identifies gaps in existing research and provides guidance for how patient-centered applications further facilitate increased transparency, AI education, and cross-disciplinary collaboration among dentists, computer scientists, ethicists, and policymakers. The future research should include clinical validation, economics, and ethical standards to make AI teledentistry use responsible and inclusive. This scoping review equips clinicians and researchers with a roadmap for responsible, patient-centered implementation of AI-enabled teledentistry, offering practical strategies and insights to enhance the quality and accessibility of remote dental care.

## SUMMARY

1. Introduction

2. Methodology

3. Results

4. Discussion

5. Conclusion

## 1. Introduction

The COVID-19 pandemic highlighted the need for remote healthcare and accelerated the emergence of teledentistry as a component of modern dental care in the past decade ^1,2^. Teledentistry leverages digital communication technologies to deliver dental services remotely in regions with inadequate or no dental facilities ^2,3^. A variety of teledentistry approaches are available, including video conferencing for in-the-moment consultations, store-and-forward techniques involving collection and transmission of diagnostic data for specialist assessment, and remote patient monitoring that enables the continuous delivery of healthcare and patient safety ^3^ .

Teledentistry has also evolved to include advanced digital technologies that have enhanced its capabilities and applications. The key technologies that positively affected teledentistry include Artificial Intelligence (AI), augmented reality (AR), three-dimensional (3D) printing, digital imaging and improved telecommunication technologies ^4,5^. AI has changed healthcare by enhancing patient care, increasing effectiveness of clinical workflow and making diagnostics and treatment planning more accurate ^6,7^. For example, AI algorithms help analyze dental images and data to assist with accurate diagnoses and effective treatment planning, thus making remote dental care delivery more precise ^7,8^.

AI-driven systems analyze large amounts of data, including dental records and imaging, to determine which treatment option is best suited to a patient ^9,10^. AI algorithms, like convolutional neural networks and deep learning models, enhance diagnostic accuracy and predictability ^9,11^. Leveraging predictive analytics, dentists can proactively treat dental issues and provide treatment plans that are tailored to specific needs of patients ^11^.

The applications of AI significantly enhanced teledentistry by improving diagnosis accuracy, tailoring treatment plans, improving operational efficiency, and increasing patient engagement and access to care ^8,12^. AI automated workflows minimizes time and cost for routine administrative and clinical work, allowing dentists to dedicate more time for patient care ^12^. It also facilitates remote monitoring and predictive analytics for better outcomes and patient satisfaction ^13,14^.

AI technologies have played a major role in facilitating the growth and acceptance of teledentistry by advancing multiple elements of dental care provision and management, including remote assessments, maintenance of patient care with less frequent in-person visits, and enhanced participation via interactive platforms and customized educational materials ^14,15^. AI-powered tools support the diagnosis and treatment of dental issues through sophisticated imaging and radiography for earlier disease detection and more precise interventions ^8,13^.

Nevertheless, there remain technological, regulatory and policy obstacles, along with privacy and ethical concerns over the use of AI in teledentistry ^3,7^. These need to be addressed so that patient-centered care can be achieved to its maximum potential.

This scoping review article discusses patient perspectives and future directions of AI-integrated teledentistry. By looking at the current status of teledentistry, the significance of AI for healthcare broadly as well as its specific implications for dental care, we hope to give insights how integration of AI into patient care could impact patients’ experience in general, and in particular for those having dental outcomes.

## 2. Methodology

In this scoping review, patient centered perspectives and future directions were reviewed in AI powered teledentistry. The study was conducted according to the Arksey and O’Malley framework and complied with the Joanna Briggs Institute (JBI) guidelines for scoping reviews in order to organize a systematic and comprehensive approach. Key contributions of the study include:

- Highlighting the relationship between AI solutions and patient experience.
- Evaluating issues related to scale, Ethics, and oversight in AI-powered remote care.
- Providing pragmatic recommendations to stakeholders, from clinicians to technologists to policymakers, on implementation challenges.

### 2.1 Eligibility Criteria

Inclusion Criteria: Peer-reviewed articles, conference papers, reviews, case studies and expert opinions published in English between 2020 and 2024 were included in the review. Selected studies focused on uses of AI in teledentistry application (i.e. diagnostics, treatment planning, remote patient monitoring) from clinical settings, in a dental practice or with patient involvement. Articles or studies were considered if it offered well-designed approaches, high-quality samples, and empirical use of AI in teledentistry.

Exclusion Criteria*:* Non-peer-reviewed articles, studies unrelated to AI in teledentistry, and articles without empirical data were excluded. The articles or studies with unclear approach, lacking empirical data, or lack of adherence to research objectives were not considered for full-text review.

Grey Literature Assessment: Conference abstracts and reports were accepted only if they contained methodological information and empirical results.

### 2.2 Information Sources and Search Strategy

This involves several cross database comprehensive research being done on PubMed, Scopus, IEEE Xplore and Google Scholar. Conference abstracts and organizational reports were also explored as sources of gray literature. The keywords used were "AI in teledentistry", "remote dental care", "AI-powered diagnostics" and "patient centered care in dentistry" and Boolean operators combining these terms. The goal of this search was to collect as much evidence as possible on the research questions.

### *2*.3 Study Selection

Articles were screened in two stages: A first step is initial screening of titles and abstracts, with a subsequent step of a full text review. Records were excluded if they were duplicate, or if the study did not meet eligibility criteria.

**Figure 1:**
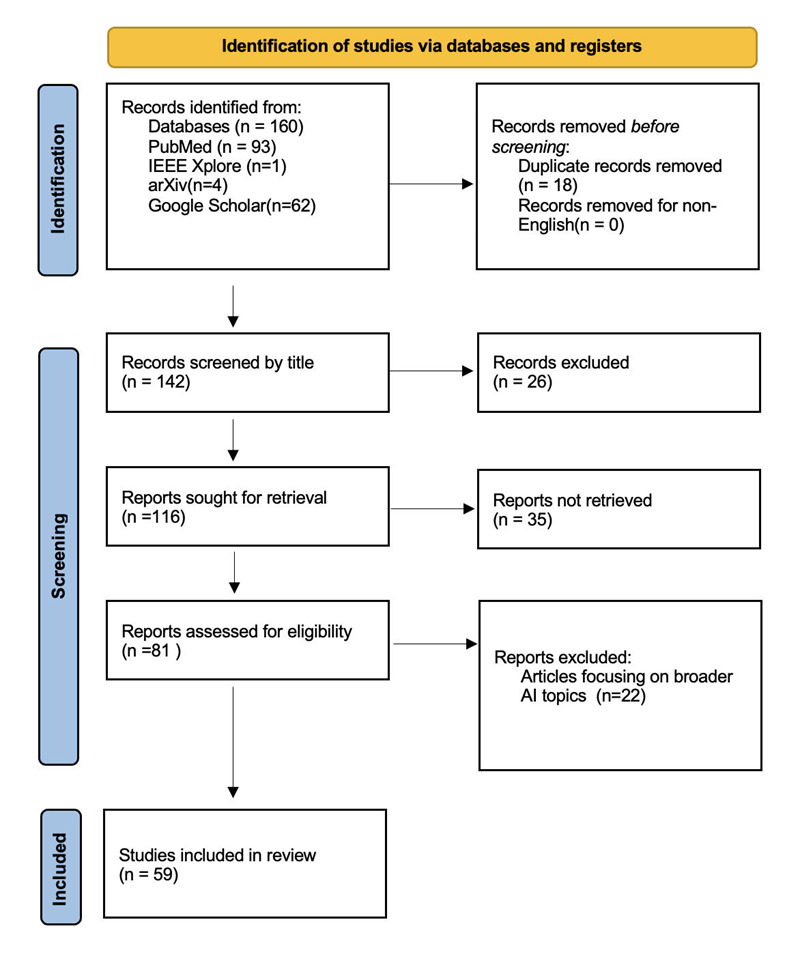
Figure 1. Study's characteristics Records were identified in databases and registers, such as PubMed, IEEE Xplore, arXiv and Google Scholar. Duplicates and irrelevant files were filtered. Of 142 titles that had been screened, 26 were removed for being irrelevant. After indexing and evaluation, 22 reports were excluded for more general AI-related issues. There were 59 studies that were taken up for the final review. The methodology follows PRISMA guidelines 60. This work is licensed under CC BY 4.0. To view a copy of this license, visit https://creativecommons.org/ licenses/ by/4.0/

From these initial searches, 160 records were found, duplicate entries and non-English studies were then excluded. Of the 142 remaining records, 26 articles were excluded on the basis of title and abstract. A full-text assessment was performed on 81 articles, and 59 articles with studies meeting eligibility criteria for inclusion in this review were selected (Figure 1).

### 2.4 Data Charting

Data were extracted using a structured data charting form, which included the following fields:

Study details: Author(s), publication year, country.

AI Technologies: Types of AI technologies used.

Clinical Applications: Diagnostics, treatment planning, remote monitoring.

Outcomes*: *Key findings, benefits, limitations, and clinical implications.

Ethical Considerations: Issues related to patient privacy and AI transparency.

### 2.5 Data Synthesis

In the synthesis process, a narrative framework was applied:

Thematic Analysis*:* The key results were sorted into technology advances, clinical use, patient outcomes, and obstacles to adoption. They were also categorized with two reviewers in order to avoid bias.

Iterative Validation*:* The thematic structure was repeated and updated through iterative checks by the research team to verify its conformance to the purpose of the study and reproducibility.

Transparency Measures: Extracted data were charted in a clear charting form for reproducibility and traceability of the synthesis.

### 2.6 Ethical Considerations

The review followed ethical standards through disclosure of the process and non-conflict of interest. Any potential study selection bias was corrected through dual-reviewer screening.

### 2.7 Protocol Registration and Amendments

This review protocol (Registration URL: https://osf.io/tpdus, Registration DOI: https://doi.org/10.17605/OSF.IO/4QD9K) was posted on the Open Science Framework (OSF) to provide methodological transparency. Any modification of the protocol was reported and explained in the final manuscript.

The review was done under the guideline of this structured methodology, it tried to provide a holistic view of how AI can aid teledentistry, including its challenges and future innovation potential.

## 3. Results

### 3.1 Motivations for Incorporating AI into Remote Dental Care

**Figure 2:**
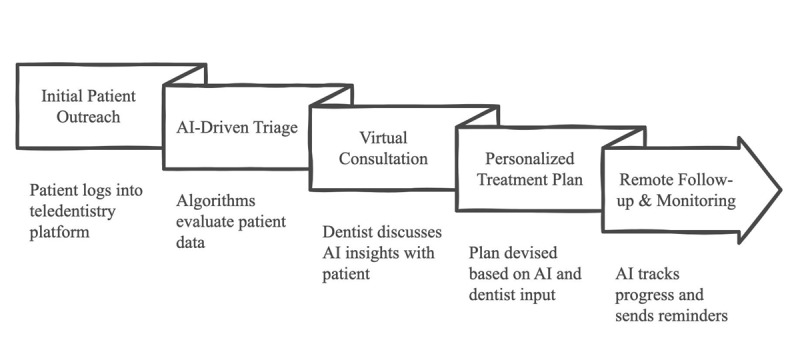
Figure 2. Patient-Centric Workflow in AI-Enabled Teledentistry This schematic outlines the stepwise process of remote dental care through an AI-assisted lens. After the patient logs into a teledentistry platform (Initial Patient Outreach), algorithms evaluate their input data to prioritize care needs (AI-Driven Triage). A virtual consultation follows, during which the dentist reviews AI-generated insights and discusses them with the patient. Based on both the dentist’s clinical judgment and AI recommendations, a personalized treatment plan is formulated. Finally, an AI-driven follow-up system tracks patient progress and sends reminders for ongoing care or further in-person assessment when necessary.

There are several reasons to integrate AI in teledentistry, but the major goal appears to improve the quality and efficiency of dental care while making it more accessible to all. This section summarizes the primary motivations for the incorporation of AI in teledentistry, like enhancing the accuracy of diagnosis and the planning of treatment, increasing the availability of dental care to underserved populations, optimizing patient outcomes and clinical workflows, and reducing costs and increasing efficiency. To demonstrate how these advantages translate into practical clinical practice, Figure 2 presents a patient-centric implementation of AI-driven teledentistry. It outlines each stage of remote care -from the initial patient outreach and AI-driven triage, through the virtual consultation and personalized treatment planning, to ongoing follow-up -emphasizing where and how AI tools help drive clinical decisions while keeping patients at the forefront.

#### 3.1.1 Enhancing Diagnostic Accuracy and Treatment Planning

One of the key drivers behind integrating AI is that it has the potential to boost the accuracy of diagnostics and treatment strategies. Dental images can be analyzed in great detail by AI algorithms, and in particular by deep learning models of the type of Convolutional Neural Networks (CNNs) ^16,17^. This then allows for earlier detection of dental issues by detecting abnormalities in radiographs, intraoral scans, and other dental imaging that sometimes can be missed ^16,17^. These AI algorithms can automatically recognize and categorize different dental conditions, such as caries, periodontal diseases, and early signs of oral cancer ^7^.

Furthermore, AI can improve the analysis of panoramic radiographs, Cone-Beam Computed tomography (CBCT), and intraoral radiography, resulting in improved identification of dental conditions for instance root fractures and periapical radiolucent lesions ^18^. Hence, AI-powered clinical decision support systems give dentists evidence-based understandings, improving the quality of decisions regarding dental care in remote settings ^7,19^. AI's predictive analytics allows proactive and tailored treatment plans for patients by taking past data as well as their characteristics into consideration ^7,19^.

#### 3.1.2 Improving Accessibility to Dental Care for Underserved Populations

AI improves accessibility to dental care, specifically for underserved populations. AI-powered teledentistry platforms narrowed the geographical gaps in access to specialized dental care by enabling remote consultations and diagnostics ^20,21^. This specifically benefits the individuals in rural or underdeveloped regions who have limited access to dental clinics. Through workflow optimization and diagnostic process automation, AI helps to reduce the cost of dental services, hence making them more affordable and accessible to underserved populations ^21^.

Additionally, AI-driven systems provide personalized oral health education and guidance, which empowers patients to better manage their oral health in underserved areas ^22^. Through these AI systems, patients can be remotely monitored on an ongoing basis, which allows for timely interventions without the need for frequent in-person visits, which is especially beneficial for patients residing in rural areas or those who have limited mobility ^20^.Furthermore, AI-based language translation and culturally attuned applications can eliminate the language and cultural barriers to dental care for diverse populations ^22^.

#### 3.1.3 Optimizing Patient Outcomes and Clinical Workflows

Optimizing patient outcomes and clinical workflows is another major driving force behind the integration of AI in teledentistry. AI strengthens organization and management of fragmented healthcare data, thereby improving record accessibility and encouraging more useful teledentistry practices ^23^. The utilization of AI in teledentistry improves patient care by allowing repetitive work and administrative procedures to be automated, leading to more efficiency and productivity ^23,24^.

AI enables continual monitoring of oral health status remotely in patients. For example, interventions can be provided in a timelier manner and do not necessitate the patients visiting the clinics, which is particularly beneficial for ongoing treatments like orthodontics ^11,24^. AI-based applications provide personalized education and training in oral health. This improves engagement of the patient in enhancing their oral health ^11^. Furthermore, it can also interpret patient data to forecast dental ailments, thus helping patients by giving timely treatment and improving overall outcomes.

#### 3.1.4 Cost Reduction and Efficiency Improvements in Dental Care Delivery

Another key motivator for integrating AI into teledentistry is cost reduction and improvement in efficiency. With AI streamlining the workflows and automating routine tasks, hence reducing operational costs for dental practices ^23,24^. This saves cost which benefits both providers and patients. Due to its accuracy, high-precision dental image analysis enhances the earlier detection and hence makes it possible for early intervention potentially reducing long-term treatment costs for patients, as early interventions often costs less than treating an advanced condition ^24^.

Besides, AI-enabled teledentistry platforms allow for remote consultations, minimizing the physical visits for many cases. This saves travel costs and time for patients, at least for those in underserved or geographically remote areas ^21^. AI based predictive analytics promote more proactive and preventative care that could lead to reduced overall costs for dental treatments in the longer term. The implementation of AI systems involves investment but the potential long-term savings together with efficiency gains provide a compelling case for integrating the systems as part of the delivery of dental care.

### 3.2 Patient-Centered Scope

#### 3.2.1 Patient Perceptions of AI in Dental Care: Trust, Acceptance, and Concerns

Patients are uncertain about the use of AI in their dental treatment. A cross-sectional regional survey posited that while some patients understood the development and advantages of AI in improving diagnostic accuracy and efficient treatment planning, others had concerns and misconceptions about it ^25^. Lack of awareness makes them have different beliefs about the application of AI to dentistry. Majority of the people are unaware of how AI is being used in dental practices. The concept still remains ambiguous for some.

Especially privacy and ethics are of high interest for patients themselves. The advent of AI created concerns for patients who are very concerned about how their personal health data is collected, used, and protected ^26^. These ethical issues cover a multitude of bases, from decision-making to accountability, and the fear that potential biases inherent in AI algorithms could impact treatment outcomes directly.

Another cause for concern is the potential changes in the dentist-patient relationship where the patients fear that with increased AI, less direct interaction with their dentists will follow, and so the level of trust and care will reduce. Furthermore, there is also the question of patient trust, about whether AI-driven diagnosis is as accurate and reliable as the traditional one. Generational impact could lead to a different acceptance threshold with younger patients being more comfortable to accept AI technologies within the healthcare system.

These concerns need to be addressed through better patient education around AI in dentistry. Providing clear and accessible information will help dispel myths and create credibility for AI applications. With the realization of the importance of AI and its advantages, it can help in gaining higher patient acceptance ^25,26^.

#### 3.2.2 AI’s Role in Enhancing Patient Engagement and Satisfaction in Teledentistry

AI improved patient engagement and satisfaction in most aspects of its encounter with teledentistry discussed here:

Personalized Patient Education*:* AI brings individualized educational resources which makes patients better understand their dental problems and corresponding treatment. Therefore, this customization is also used to facilitate informed decision making and enhance patient engagement ^27^.

Virtual Consultations*:* The AI-driven virtual consultations will be made more accessible for remote patients. Barriers could be reduced and improve convenience and patient satisfaction ^27^.

Language Translation*:* Language barrier between the patients and dentists may be effectively overcome with the help of AI translation tools which comes with effective translation and improved patient experience ^27^.

Virtual Reality Simulations*:* AI based virtual reality and dental processes support simulation reduces patients' anxiety and improves overall experiences with dentistry ^27 .^

Chatbots and Virtual Assistants: AI chatbots facilitate real time responses to patient queries and personal interactions that increase patient engagement and satisfaction ^28 .^

Improved Efficiency*:* Using AI can help to decrease the queue and improve appointments making patients satisfied ^28.^

Since the latter will provide a patient centered care and ultimately a more satisfied patient and stronger dentist-patient relationship ^7,27^.

#### 3.2.3 The Impact of AI on Care Continuity and Follow-up in Remote Dental Services

AI positively influences the continuity of care and follow-ups that happen in the remote dental service, as it leads to the following:

Enhanced Remote Monitoring: AI helps dentists monitor their patients’ oral health remotely so that they can identify issues that need further treatment without visits^7.^

Personalized Treatment Planning*:* The use of AI in treatment makes it possible to tailor care plans and ensure that future interventions are customized based on the needs and progression of each patient, thereby ensuring continuity of care ^7^.

Predictive Analytics*:* AI predicts the potential problem of oral health and helps the dentist to predict and plan for preventive follow-up procedures ^7^.

Improved Patient Engagement: AI enables patient compliance with follow-up care plans by communicating and training the patient individually, leading to long term engagement ^7,11^.

Efficient Referral Systems*:* AI optimizes referrals to specialists as needed, ensuring appropriate and prompt follow-up ^11^.With limited access to dental services, AI-driven teledentistry offers a model of sustaining continuity of care for underserved populations with potential for narrowing oral health access disparities ^11,29^.

#### 3.2.4 Strategies for Improving Patient-Centered AI Applications in Teledentistry

To enhance patient-centered AI applications in teledentistry, the following strategies may be helpful:

Patient Education: Develop patient education courses on AI in dentistry, helping patients overcome fears and misconceptions^25,26.^

Transparency and Communication*:* The implementation of AI in patient care will provide transparency and discussions regarding its implementation and data management, thus creating trust ^26,27^.

Personalization: Use AI to deliver a personalized service, including tailored treatment plans and patient resources, based on the needs and preferences of the individual patient ^7,30^.

Enhanced Data Security: Establish proper data protection policies to eliminate privacy issues and ensure the privacy of patient data ^26^.

Feedback Mechanisms: Including the feedback from patients while building AI systems for better usability and patient satisfaction ^27^.

Training for Dental Professionals: Train Dentists on using AI to provide better care to patients and maintaining the dentist-patient relationship ^7^.

Addressing Ethical Considerations: Establish ethical frameworks for AI deployment in teledentistry that ensure responsibility, reduction of bias, and autonomy for patients ^26^.

**Figure 3:**
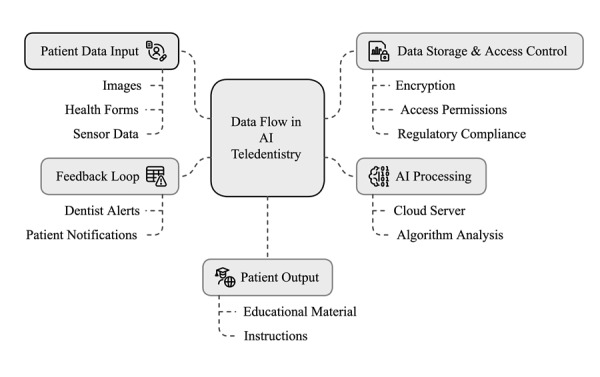
Figure 3. Data Flow and Privacy Considerations in AI-Enabled Teledentistry The schematic depicts how patient data (images, health forms, sensor data) travel from initial collection to AI processing on a secure cloud server. Data storage and access control measures (encryption, permissions, regulatory compliance) ensure confidentiality and adherence to privacy standards. A feedback loop provides real-time alerts and notifications to both dentists and patients, while the patient output node delivers personalized educational materials and instructions, closing the data cycle in a patient-centric manner.

These strategies can help AI tools to get the most out of teledentistry to ensure it is more patient-focused, better care, better patient satisfaction, and better health outcomes. Figure 3 further illustrates how the data flow and privacy considerations underpin these strategies, demonstrating the crucial steps for secure data handling - from patient input and AI processing to secure storage and feedback loops that maintain trust and engagement.

### 3.3 Future Directions and Research Opportunities

#### 3.3.1 Unexplored Areas and Emerging Trends in AI and Teledentistry

Use of AI in teledentistry is a new and emerging field with plenty of unexplored areas and trends whose potential has yet to be researched. Personalized treatment planning is one of the most recent applications of AI in teledentistry. AI can be used to optimize periodontal disease management and the treatment outcomes of patients suffering from complex systemic conditions like diabetes, as suggested by recent studies ^31^.

Another emerging area is how Large Language Models (LLMs) can be used to generate AI personas to simulate patients. Although explored initially in marketing research ^32^, this could also be extended towards the simulation of patient behaviors relevant to dentistry for better patient education and treatment planning in teledentistry.

The developments in Generative AI models can help to improve the quality of dental imaging and remote diagnostics, for example by bettering image analysis for improved accuracy and efficiency in remote dental assessments ^33^. Furthermore, AI-based predictive analytics which is, albeit currently prominent in agriculture ^34^, could find an excellent application in teledentistry for prediction of dental diseases and optimization of preventive care strategies.

In relation to dental software and tools, AI can also potentially enhance the delivery of dental software and the applications available to the dentist. Given AI’s potential impact on software engineering, it is plausible that AI-enabled dental software can be developed to make remote delivery of dental care possible ^35^. AI’s potential to enhance cognitive reasoning can be amplified by incorporating AI into systems to promote interdisciplinary approaches. Extrapolating this idea further, it is easy to imagine that AI can bolster both telemedicine and teledentistry through forming collaboration between general practitioners from different sectors, which is key to effective delivery of oral health care.

Advanced AI techniques, for signal and image processing, have found direct relevance in teledentistry. Investigation in these directions could upgrade the quality and interpretation of dental images and videos exchanged during remote consultations ^36^. In addition, the use of AI-based diagnostic algorithms developed for other medical fields, such as identification of thyroid-associated ophthalmopathy from photographs of a person's face ^37^, outlines the potential of AI in teledentistry to identify the oral signs of systemic diseases.

#### 3.3.2 Research Gaps and Suggested Studies to Advance the Field

Despite all this development, a few research gaps in the literature of AI in teledentistry are:

Limited Studies on Teledentistry-Specific AI Applications: Sparse number of studies have targeted AI applications for teledentistry-specific functions of delivering dental care remotely. This indicates that studies considering unique challenges and opportunities in teledentistry are warranted ^38^.

Lack of Large-Scale Clinical Validation: While some AI models have already received certain regulatory approvals, for example, the FDA- approved AI model for diagnosing periodontal disease ^39^, still there is a striking lack of large-scale clinical studies that could support the efficacy of AI applied to different aspects of teledentistry.

Integration with Existing Platforms: There are a handful of studies on the best practices for integrating AI into current teledentistry platforms and workflows. Considerably more research is needed to advance the development of integration methodologies that will ensure seamless care provision ^40^.

Patient Outcomes and Experiences: Further comparative research is necessary to determine whether AI-driven teledentistry is associated with better patient outcomes and experiences than conventional in-person dental care that do not employ AI applications ^41^.

Cost-Effectiveness Analysis: Limited research exists on the economic impact of implementing AI in teledentistry. Cost-benefit analyses are necessary to inform stakeholders and guide investment decisions ^42^.

Ethical and Privacy Considerations: Ethical dilemmas and challenges to privacy in the use of AI in remote dental care need thorough exploration to establish that the handling of patient data is conducted in a secure and ethical manner.

Future studies should focus on addressing these gaps such as conducting large-scale clinical trials, introducing integration frameworks, assessment of patient outcome, economic analyses, and even formulating guidelines for the use of ethical AI in teledentistry.

#### 3.3.3 The Role of Interdisciplinary Collaboration in Advancing AI in Teledentistry

Advancing AI in teledentistry necessitates interdisciplinary collaboration across various fields:

Computer Science and Dentistry: Computer scientists should collaborate with dental specialists in order to facilitate timely development of AI algorithms that diagnose and plan treatment through image analysis ^43^.

Ethics and AI: Incorporating ethical frameworks into AI will translate to responsible deployment of AI, considering its bias, transparency, and fairness in dental care ^44,45^.

Data Science and Privacy: The collective efforts by data scientists and privacy specialists are required in managing patients' data securely and ethically within the scope of the existing relevant regulation for data protection ^46^.

Human-Computer Interaction (HCI): HCI specialists may enable the usability of AI-based teledentistry platforms and accordingly helping in proper adoption by dentists and patients ^47^.

Psychology and AI: Psychologically informed AI is envisioned to contribute toward development of such AI systems that better comprehend the patient behaviors around anxiety and engagement ^48^.

Education and AI: Educators can develop training programs for dental professionals and patients to practice on how best to use AI in teledentistry ^49^.

Law and Technology: Legal experts can assist in developing regulatory frameworks to address liability, consent, and cross-border practice in AI-driven teledentistry ^50^.

Economics and Healthcare Management: Economists may carry out cost-effectiveness research in the AI implementations that will guide investment and policy formulation ^46^.

Software Engineering and Dentistry: The collaboration in software development brings teledentistry robust, customized AI solutions that are better in functionality and user experience ^44^.

Linguistics and AI:Linguists could refine AI-powered communication tools, thereby enabling better patient provider interaction in multilingual contexts ^47^.

Interdisciplinary collaboration will further enhance AI development and teledentistry applications, ensuring that technological advancements will meet clinical needs, ethical standards, and user expectations. Such diverse expertise enables this field to overcome the associated complex challenges of developing more effective and responsible AI solutions for enabling remote dental care.

## 4. Discussion

The future of teledentistry AI could revolutionize dental patient remote care by enhancing diagnostic accuracy, increasing care in underserved communities, maximizing patient outcomes and decreasing costs. Deep learning algorithms (e.g., convolutional neural networks) trained on dental images have been reported to enhance diagnosis for dental conditions that dentists would otherwise miss^16,17. ^The AI can not only be used for detection but also to support personalized treatment planning that will likely be available as data science and artificial intelligence come together to enable predictive analytics, and based on past observations and patient attributes ^7,19^.

AI-driven teledentistry facilitates remote diagnostics and consults in the most remote locations of the world, like villages and remote regions ^21,22^. With the automation of routine tasks and streamlining workflows, AI can boost clinical productivity for dentists, giving them more time to focus on individual care ^23,24^. These changes may help patients to become more involved and satisfied, with AI providing personalized education, virtual consultations, and appointment scheduling ^7,28^.

### 4.1 Key Findings and Contributions

Diagnostic and Clinical Applications:The analysis confirmed that AI algorithms, in particular machine learning and deep learning algorithms, are excelled in studying dental images and identifying caries and periodontal disease. These technologies can also be used to remote monitor patients and provide more precise diagnoses in places that resource-limited or underserved regions. Notably, there were several research studies that show AI-based workflows can significantly cut clinicians’ time on the routine tasks and let them focus more on the patients care.

Patient-Centered Insights:A core focus of this review was on patients’ experience, acceptance and ethical aspects of AI application. Data showed that although most patients like having the ability to receive their dental services at home, there were still issues with data security, accuracy, and fewer face-to-face interactions. It is recommended that establishing clear communication on how data would be handled, the adoption of intuitive AI-driven platforms, and a continued role for human dentists in final decision-making were suggested to improve trust and acceptance.

Regulatory and Ethical Considerations:The data also highlighted the need to establish explicit regulatory pathways for AI in teledentistry. Ethical challenges– algorithmic bias, fair access, data privacy – were common obstacles to adoption. These concerns require dentists, AI engineers, data privacy professionals and policymakers to work collaboratively on rules to ensure patient safety and trust.

Future Directions and Research Gaps: Despite increasing applications of AI, large-scale clinical validation studies are limited. Cost-benefit analysis, longitudinal studies of patient outcomes and comparative trials against standard diagnostic methods are needed. Additionally, there is the next-generation AI (e.g., Large Language Models, generative algorithms) that can further develop teledentistry services, but will require robust ethical frameworks and ongoing stakeholder engagement.

Implications for Practice: Through a synthesis of contributions from a diverse source, this review explains how AI can have a useful role to play in teledentistry to enhance diagnostic accuracy, clinical workflow, and reduce healthcare disparities. Moreover, it underscores that technological innovation cannot be made without rooted in patient-centric values - trust, transparency, and ensuring equitable access to remote dental care.

### 4.2 Gaps and Opportunities

However, some research gaps and challenges remain. Large-scale, clinical validation of the efficacy of AI applications used in teledentistry (which is essential for regulatory approvals and wider acceptance) is still lacking ^39.^ Data privacy and ethical issues, as well as the risk of bias in AI algorithms, still pose a challenge ^26.^ Therefore, it requires more research on secure data management, and development of an ethical code for use of AI in remote dental care to ensure patient trust and acceptance.

There is also great scope for unexplored areas, such as employing large language models (LLMs) to simulate a patient interaction in order to improve patient education ^32^ or to increase the resolution and accuracy of dental imaging using generative AI models for remote assessment ^33^. A closer collaboration between computer scientists, practitioners, ethicists and other stakeholders will be key to take AI in teledentistry to the next level ^43,44^.

### 4.3 Strengths and Limitations

This review presents an array of advantages of integrating AI and teledentistry while admiring the cutting-edge technologies and increased benefits to patient care. The advantage of AI and dental telecare is emboldened by the prospect of extending remote dental services to underserved populations. The drawbacks include relying on the past literature to reflect today's developments in this continuously evolving area which may preclude broader discussions about the topics or studies that emerged more recently.

### 4.4 Comparison with advancements in similar field

Using comparative studies with other medical specialties that have already successfully been integrated into remote services such as telemedicine can also help to guide teledentistry. Yet teledentistry, which, unlike other medical specialties, requires the tactile exam and range of oral health scenarios, requires AI solutions built for it ^38^. The literature indicates that the particular difficulties of teledentistry demand teledentistry-focused AI studies.

The Table 1 demonstrates how AI’s core capabilities (image analysis, remote consulting, and patient engagement) are used in teledentistry as compared to other healthcare areas ^51-60^. It offers overlapped benefits (improved accuracy, streamlined workflows) and unique issues in teledentistry. The table also underscores the shared lessons learned across healthcare fields that can support the development of AI-enabled teledentistry moving forward.

**Table 1:** Table 1. AI applications in teledentistry versus Other AI-intergrated healthcare fields ^51-60^ Overview of different aspects of AI applications in teledentistry compared to other healthcare domains, highlighting similarities and differences in their implementation, benefits and challenges.

Aspect	AI in Teledentistry	AI in other Healthcare Fields
Diagnostic Tools	- AI-powered predictive models for oral health risk assessment - Machine learning algorithms for dental disease diagnosis - AI-assisted screening of dental radiographs ​	- AI tools for ENT diagnostics - Convolutional neural networks for image analysis in various specialties - AI-based disease prediction systems ​
Remote Consultation	- Smart device-based remote screening and diagnosis - AI-enabled triage systems for dental patients​	- Telemedicine platforms with AI- assisted diagnosis - Virtual health assistants for initial patient assessment ​
Patient Engagement	- AI-powered self-diagnosis tools for early-stage dental issues - Personalized oral health education apps ​	- AI chatbots for patient education and symptom checking - Personalized health recommendations based on AI analysis ​
Data Acquisition & Quality	- Integration of digital dentistry tools for data collection - AI-enhanced record keeping systems ​​	- AI algorithms for processing structured and unstructured healthcare data - Natural language processing for medical records analysis ​​
Ethical & Regulatory Considerations	- Need for data privacy and security in teledental applications - Regulatory clarity required for AI integration in dental practice​	- Concerns about AI tool accuracy and reliability - Need for regulatory frameworks to ensure patient safety ​​
Scalability & Accessibility	- Potential to serve larger and underserved populations in remote areas - Improved access to dental care through AI-powered teledentistry​​	- AI tools enabling broader access to specialized medical expertise - Potential for reducing healthcare disparities ​​
Implementation Challenges	- Integration with existing dental practice systems - User-friendliness of AI tools for dental professionals​	- Automation bias and over- reliance on AI diagnostics - Need for healthcare professional training in AI tool use ​​

### 4.5 Integrating AI into Existing Teledentistry Practices

AI adoption within the existing teledentistry systems must confront technical, human and systemic challenges to adoption. The following section highlight some of the major challenges and offer practical approaches for a patient-centric, ethical, and cost-efficient shift towards AI-based teledentistry.

The Table 2 illustrates practical recommendations for overcoming the challenges of AI implementation into current teledentistry systems from the standpoint of technical integration, workforce readiness, patient issues, ethics, cost and long-term scalability.

## 5. Conclusion

AI in teledentistry has the potential to transform dentistry or dental care through increased diagnostic accuracy, faster clinical outcomes and better access to treatment by marginalized populations. By using AI technologies like deep learning, predictive analytics, and treatment planning, teledentistry can provide effective, personalized dental treatment at lower prices, and satisfied patients. Beyond overcoming the barrier to universal dental care, these innovations create new possibilities for preventive oral care.

However, the full potential of AI in teledentistry can be realized once the challenges - ethical issues, privacy concerns, and lack of clinical research and evidence - are overcome. Addressing challenges requires collective effort by dentists, computer scientists, policymakers and ethicists to create safe,

**Table 2:** Table 2. Barriers and actionable solutions to integrating AI into existing teledentistry Presents actionable solutions to overcome the barriers in integrating AI into existing teledentistry practices, addressing key aspects such as technical integration, workforce readiness, patient concerns, ethical considerations, financial constraints, and long-term scalability.

Barrier	Solution
Technical Integration	- Build standardized APIs for integration with pre-existing dental systems - Use cloud-based AI solutions to minimize on-premises hardware requirements - Work with dental software
Workforce Readiness and Training	- Offer extensive training programs for dental professionals on AI tools and applications - Incorporate AI education into dental school curriculum - Provide continuous professional development courses on AI in dentistry
Addressing Patient- Centric Concerns	- Implement strong data privacy and security controls - Develop user-friendly interfaces for patient interaction with AI tools - Educate patients on the benefits and limitations of AI in teledentistry
Ethical and Regulatory Alignment	- Collaborate with regulatory bodies to define clear guidelines for AI use in teledentistry - Develop ethical frameworks for AI implementation in dental practice - Ensure transparency in AI decision- making processes
Financial Constraints and Cost- Effectivenes	- Provide flexible pricing models for AI solutions, like subscription-based options - Exhibit ROI through improved efficiency and patient outcomes - Get partnerships with dental insurance providers to cover AI-assisted treatments
Scalability and Long- Term Maintenance	- Build modular AI systems that can be easily updated and scaled - Institute ongoing support and maintenance contracts with AI providers - Implement feedback loop for continuous improvement of AI algorithms

effective and patient-centered systems. Additionally, more research is required to confirm how AI can be used in practice, optimize cost, and to establish and enforce ethics for AI use.

The success of AI-enabled teledentistry will depend on its ability to deliver affordable, quality dental treatment to people around the world. Using cutting-edge technologies and patient-centric approaches, the industry can advance and transform oral care. More research and collaboration will improve the ethics, clinical effectiveness, and social desirability of AI in teledentistry.

This scoping review provides clinicians and researchers with the tools to critically consider and implement AI-enabled teledentistry for responsible, patient-centered implementation. With practical strategies and highlighting of potential pitfalls, the review provides the clinical and scientific community with a road map for improving the availability and quality of remote dentistry.
